# Relationship between Prognostic Nutritional Index and Amputation in Patients with Diabetic Foot Ulcer

**DOI:** 10.3390/diagnostics14070738

**Published:** 2024-03-29

**Authors:** Belgin Coşkun, Müge Ayhan, Serap Ulusoy

**Affiliations:** 1 Infectious Diseases and Clinical Microbiology, Ankara Bilkent City Hospital, 06800 Çankaya, Turkey; dr.mugeayhan@hotmail.com; 2 General Surgery, Ankara Bilkent City Hospital, 06800 Çankaya, Turkey; serapulusoy13@gmail.com

**Keywords:** amputation, diabetic foot, prognostic nutritional index, infection, inflammation markers

## Abstract

The prognostic nutritional index (PNI) is a new marker used to assess a patient’s nutritional and immune status. It is calculated using serum albumin levels and total lymphocyte count. The aim of this study was to investigate the relationship between PNI and amputation in patients with diabetic foot ulcer (DFU). Patients with DFU were enrolled in this retrospective study. In our study, a total of 386 patient data, of 110 (28.5%) amputated and 276 (71.5%) non-amputated patients, were statistically analyzed. PNI values were significantly lower in the amputated patient group than in the non-amputee patient group (*p* < 0.001). According to the ROC analysis results, PNI was significant in the prediction of amputation at an excellent level (AUC = 0.937 (0.911–0.963), *p* < 0.001). The optimal cut-off point for PNI was found to be 39,005. There was classification success for this cut-off point: sensitivity was calculated as 82.7% (74.1–89) and specificity as 93.1% (89.3–95.7). In the multivariate model, the odds ratio (OR) (95% CI) was calculated as 81.8 (38.5–173.7) for PNI. The PNI was associated with an increase in amputation rate in patients with DFU. By using PNI, patients can be directed to advanced centers and have access to early and appropriate interventions.

## 1. Introduction

Globally, 537 million people are affected by diabetes. It is thought to reach 783 million in 2045 [1]. Diabetes Mellitus (DM) is a disease with high mortality and morbidity. The increasing prevalence of DM in the world means that diabetes mortality and morbidity are also increasing. According to World Health Organization, 1.5 million deaths are directly attributed to diabetes each year. Diabetic foot ulcer (DFU) and DFU-related amputation are strongly associated with morbidity in diabetic patients [2]. In a study conducted with 8905 DM patients, the incidence of foot ulcer development was 5.8% after 3 years of follow-up. In these patients, the rate of osteomyelitis was 15% and the rate of amputation was 15.6% [3]. In a recent study by Aydın et al. conducted in our country, the amputation rate was found to be 18.8% in DFU [4]. DFU continues to be a big problem in our country as well as in the World. Many factors play key roles in the development of diabetic foot ulcer (DFU) such as peripheral neuropathy, peripheral vascular disease, poor glycemic control and foot trauma [3]. Infection of ulcers and peripheral vascular disease can result in osteomyelitis and amputation [5]. Living with an amputated limb causes many social and economic problems. Controlling preventable risk factors for amputation in patients followed up with DM and early treatment of patients at high risk is very important. Peripheral vascular disease (PVD), neuropathy, hyperglycemia, advanced age, infected DFU, osteomyelitis are risk factors for amputation [2]. Recently, various cost-effective biomarkers such as prognostic nutritional index (PNI), systemic immune inflammatory index (SII), neutrophil lymphocyte ratio (NLR), platelet lymphocyte ratio (PLR) have been used to predict outcomes of different inflammation-related disorders [6,7,8]. In the follow-up of diabetic patients, the nutritional status of the patients is as important as inflammation [9]. The PNI is calculated using two main components: albumin levels and peripheral lymphocyte count. For this reason, both inflammation and malnutrition can be evaluated together with PNI. Studies have determined the relationship between PLR, NLR, SII in DFUs and amputation [6,7]. But there has not been any study showing the relationship between amputation and PNI. We know that high inflammation and malnutrition are seen much more frequently in diabetic patients than in nondiabetic patients [10,11]. We aimed to show the relationship between PNI and amputation with this study. 

## 2. Materials and Methods 

### 2.1. Patient Population

Our study was a retrospective observational study. An experienced team consisting of a doctor and three nurses works in wound care in the chronic wound unit of our hospital. In our study, patients with DFUs who were followed up in the chronic wound unit of the our hospital between January 2021 and January 2022 were screened. Patients who did not have regular follow-up records in the chronic wound unit or who did not have complete blood count (CBC) and biochemistry tests at their first admission to hospital and under 18 years old were excluded from the study. Patients’ demographic data such as age, gender, history of comorbidity, and history of osteomyelitis, were obtained from hospital medical records.

### 2.2. Biochemical Analysis

Patients laboratory values taken at the first admission to the hospital, such as CBC, serum albumin levels, blood glucose level, glycated hemoglobin (HbA1c), kidney function test, C-reactive protein (CRP), erythrocyte sedimentation rate (ESR), NLR, PLR, SII and PNI, were recorded. SII was calculated using the formula (SII = platelet count × neutrophil count/lymphocyte count). PNI was calculated using the formula ((10 × albumin) + (0.005 × peripheral lymphocyte count (/mm^3^)). 

### 2.3. Clinical Follow-Up

Patients who applied with DFU were followed up for 6 months. During this process, the patients were always evaluated by the same team and the decision for amputation was made. At the end of 6 months, the amputation status was recorded. Patients were classified as major or minor amputation status. Major amputation was defined as amputation of a portion proximal to the ankle joint. One hundred and one amputated patients had minor amputations; nine patients had major amputations. Since dividing into groups would not be statistically significant, we did not divide them into major or minor amputations. We divided them into amputee and non-amputee groups.

The demographic characteristics, clinical outcomes, biochemical parameters, comorbidities, inflammation markers such as NLR, CRP, SII, PLR, and ESR, and the PNI values of the two groups were compared. The impact of the PNI values on amputation risk of diabetic patients with DFU was investigated. 

### 2.4. Statistical Methods

The statistical analysis of the data was performed using SPSS software (Version 22, SPSS Inc., Chicago, IL, USA). Frequency (n) and percentage (%) were used to report descriptive statistics for categorical data. Descriptive statistics for continuous data were reported as mean ± standard deviation (SD) or median (interquartile: Q1–Q3) based on the assumption of normal distribution. To assess the normality assumption of numerical data, the Kolmogorov–Smirnov test was used along with Histogram and Q-Q plots. Levene’s test was used to test the assumption of homogeneity of variances. When the assumptions for parametric test were met, the Student *t*-test was used to compare continuous data between two independent groups, and when the assumptions were not met, the Mann–Whitney U test was employed. The effects of the patients’ PNI and SII scores on predicting amputation was investigated using Receiver Operating Characteristic (ROC) analysis. ROC curves and the Area Under the Curve (AUC) were calculated along with 95% confidence intervals. AUC values were interpreted as follows: 0.9–1: excellent, 0.8–0.9: good, 0.7–0.8: fair, 0.6–0.7: weak, and 0.5–0.6: poor. The Youden index (maximum sensitivity and specificity) was used to determine the optimal cut-off point in the ROC analysis. The performance of cut-off points was evaluated using sensitivity, specificity, positive predictive value (PPV), negative predictive value (NPV), and positive likelihood ratio (LR+) values. Risk factors affecting amputation prediction were determined by univariate and multivariate binary logistic regression analyses. Odds ratios (ORs) were calculated for each statistically significant parameter in both univariate and multivariate models, along with 95% confidence intervals. For all comparisons, the statistical significance level was accepted as *p* < 0.05.

### 2.5. Ethical Approval

The study was approved by the ethics committee of Ankara Bilkent City Hospital. The approval date and number were 6 September 2023, and E1-23-3944, respectively.

## 3. Results

In our study, a total of 458 patients were evaluated retrospectively. Twenty-two patients were excluded from the study because they were under the age of 18 and 50 patients were excluded due to missing data. Finally, a total of 386 patients’ data, from 110 (28.5%) amputated and 276 (71.5%) non-amputated patients, were statistically analyzed. The mean age of the patients was 59.62 ± 13.97. There were 241 (62.4%) males and 145 (37.6%) females in our study. 

The comparison of demographic and clinical characteristics between two groups are presented in Table 1.

The mean age of the study groups was not significantly different (*p* = 0.101). The mean age was 57.77 ± 13.08 years in the amputee group and 60.36 ± 14.26 years in the non-amputee group. The rates of smoking, comorbidity, rheumatological disease, coronary artery disease, peripheral neuropathy, chronic renal disease, venous insufficiency, chronic heart failure and osteomyelitis were not significantly different between the groups (Table 1). There were no patients in either group with chronic liver disease. The incidence of arterial thrombosis and venous thrombosis in the amputated patients group was significantly higher than in the non-amputees patient group (*p* = 0.040, *p* = 0.009, respectively) (Table 1). The comparison of laboratory blood values and some prognostic indicator values between two groups are presented in Table 2.

White blood cell (WBC), neutrophil, CRP, glucose, and HbA1c values were significantly higher in the amputated patient group than in the non-amputee patient group (*p* = 0.002, *p* = 0.003, *p* = 0.035, *p* = 0.021, *p* = 0.015, respectively). Albumin and PNI values were significantly lower in the amputated patient group than in the non-amputee patient group (*p* < 0.001, *p* < 0.001, respectively). Lymphocyte, platelet, NLR, PLR, urea, creatinine, ESR and SII values were not significantly different between the groups (Table 2). The AUC, sensitivity, specificity, positive–negative predictive and positive likelihood ratio values demonstrating the success of the Prognostic Nutrition Index (PNI), and albumin in amputation prediction presented in Table 3. There was no statistically significant difference between the AUC values of albumin and PNI parameters in amputation prediction (*p* = 0.957).

**Figure 1:**
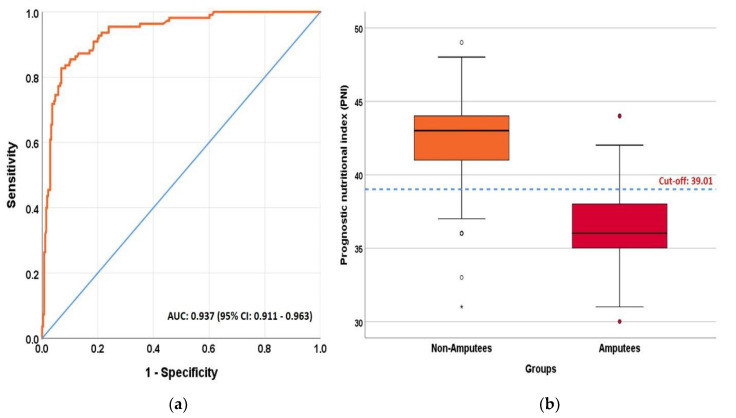
(**a**) ROC curve for prognostic nutritional index (PNI) values to predict amputation; (**b**) Box-plot showing the distribution of prognostic nutritional index (PNI) values among research groups.

According to the ROC analysis results, PNI was significant in the prediction of amputation at the excellent level (AUC = 0.937 (0.911–0.963), *p* < 0.001) (Table 3). The optimal cut-off point for PNI to predict amputation in patients with DFU was found to be 39,005. Classification success for this cut-off point; sensitivity was calculated as 82.7% (74.1–89) and specificity as 93.1% (89.3–95.7). The optimal cut-off point for albumin to predict amputation in patients with DFU was found to be 38.5. Classification success for this cut-off point; sensitivity was calculated as 81.8% (73.1–88.3) and specificity as 93.1% (89.3–95.7). Additionally, there was no significant difference between AUC values (*p* = 0.957), but sensitivity, PPV, NPV and LR + values of PNI were higher than the predictive values of albumin (Table 3). The ROC curve plot of the PNI values and the box-plot showing the distribution of PNI values among the study groups are presented in Figure 1.

The results of univariate and multivariate binary logistic regression analyses performed to determine the risk factors for amputation are presented in Table 4. 

Venous thrombosis, arterial thrombosis, WBC, neutrophil, CRP, HbA1c, and PNI were found to be significant in the univariate analysis (*p* < 0.05 for all comparisons, Table 4). These variables, which were found to be significant in the univariate model, were included in the multivariate model. Albumin and glucose, which were found to be significant as a result of basic statistical analyses, were not included in the multivariate model, because albumin was used in the calculation of the PNI index and glucose was highly correlated with HbA1c. Venous thrombosis, WBC, neutrophil, and CRP had no significant effect on amputation in the multivariate model (*p* > 0.05 for all comparisons, Table 4). Arterial thrombosis, HbA1c, and PNI were found to be a risk factor for amputation by multivariate binary analysis (*p* = 0.014, *p* = 0.022, *p* < 0.001, respectively). In the multivariate model, the OR (95% CI) was calculated as 3.04 (1.25–7.37) for arterial thrombosis, 1.24 (1.03–1.48) for HbA1c, and 81.8 (38.5–173.7) for PNI (Table 4). Patients with the PNI value less than 39,005 were 81.8 times more likely to be amputated than those with a PNI greater than 39,005.

## 4. Discussion

This study showed that low PNI was associated with higher amputation rate in DFUs. The PNI is used to assess the relationship between immune response and nutritional status [12]. The PNI is calculated using two main components: serum albumin levels and total lymphocyte count. In the studies, low albumin was found to be significantly associated with major amputation and mortality, similar to our study [13,14]. Our results showed that the mean serum albumin value was 43 gr/L in non-amputee group and 36 gr/L in amputee group. Albumin was significantly lower in the amputated patient group than in the non-amputee patient group (*p* < 0.001). Serum albumin is a protein that synthesized by liver. In our study, there was no statistically significant difference between the groups in chronic renal disease, which may cause albumin loss. There were no patients in either group with chronic liver disease that would affect albumin synthesis. Exudation and debridement in the wound cause large amounts of protein loss. At the same time, protein is needed for tissue healing [13]. If the protein synthesis rate is not sufficient, tissue healing slows down. Albumin is commonly used as a marker of a patient’s nutritional status [15]. However, in diabetic foot patients, it is associated with disease severity as well as malnutrition [13]. During bacterial infection, these patients often have leukocytosis, neutrophilia, and lymphopenia. It also decreases in albumin, which is a negative acute phase reactant. So evaluating albumin independently of infection could lead to incorrect results. For this reason, PNI, which evaluates inflammation and nutritional status together, was preferred. In the PNI formula, low serum albumin values contribute to a low PNI score, indicating poor nutritional status and high disease severity [15,16].

Malnutrition is an important nutritional problem in DM patients as well as obesity [11]. Studies have shown that the underlying cause of malnutrition in DM patients is impaired insulin secretion [9]. In one study, one in every seven type 2 diabetes patients followed up was found to be at risk of malnutrition [11]. Macrovascular complications are more common in DM patients with malnutrition [17]. It has been shown that hypertension and hyperlipidemia are more common in patients with malnutrition. Hypertension and hyperlipidemia increase microvascular and macrovascular complications in DM patients [11,18]. It is known that vascular damage increases the risk of amputation in patients followed with DFU [5]. For this reason, malnutrition is associated with amputation in patients with DFU. At the same time nutrition plays an important role in regulation of immune response. So protein energy malnutrition is associated with immunodeficiency [19]. Therefore, albumin, a component of PNI, is important in predicting the risk of amputation in DFUs by predicting malnutrition.

The other main component of PNI is lymphocyte count. Lymphocytes are a type of WBC and critical part of the immune system. Total lymphocyte count in the blood can reflect the immune function. A low lymphocyte count can indicate immune system suppression or disfunction [20]. In our study, although there was no statistical difference between the amputee and non-amputee groups, the lymphocyte count was lower in the amputee group. Similar to our study, in studies evaluating the relationship between PNI and mortality in infective endocarditis and Crimean–Congo hemorrhagic fever, PNI was found to be significant, although there was no significant difference in lymphocyte count between the groups [8,21]. In the PNI formula, lower lymphocyte counts contribute to a lower PNI score, indicating potential immune system compromise. But while there is no statistical difference between the groups in lymphocyte counts, PNI is significantly lower in amputee patients. This is important to highlight malnutrition.

In our study, the demographic and clinical characteristics of the amputee and non-amputee groups were similar, but the incidence of arterial and venous thrombosis was higher in the amputee group. In the literature, peripheral arterial disease was higher for the amputee group [22]. Peripheral artery disease is present in almost half of patients with diabetic foot ulcers [23]. Peripheral arterial disease is a one of the important risk factors for healing failure and amputation in patients with DFU [24]. Similarly, in our study, arterial thrombosis increased the risk of amputation 3.04 times.

Studies have shown that poor sugar regulation both increases infections and impairs tissue healing [5,25]. The glycosylation end products formed as a result of hyperglycemia in DFUs cause the release of cytokines and the formation of reactive oxygen species (ROS). This causes inflammation in the tissue and delays tissue healing [25]. In our study, the amputee patients had poor glucose regulation and had elevated levels of HbA1c.

Although not significant in multivariate analysis, wbcs, neutrophils and CRPs, which are indicators of infection, were high in amputee patients. Infections are the most common factors causing amputation in DFUs [26]. A prospective study reported that only 46% of infected DFUs recovered [27].

Increased inflammation is a poor diagnostic factor for DFU. Therefore, there have been studies showing the relationship of NLR, PLR, SII with amputation and osteomyelitis in diabetic foot patients [4,28]. In our study, there was not found to be significantly higher NLR, PLR and SII levels in the amputee group. PNI, another inflammation marker, was significantly lower in the amputation group. Although there was no difference in lymphocyte count between the groups, the PNI was low in the amputation group. This demonstrated the importance of albumin. When the ROC analysis findings for albumin were compared with the PNI ROC analysis findings, we determined that there was no statistically significant difference between the AUC values (*p* = 0.957), and the AUC values were almost the same. Additionally, there was no significant difference between AUC values, but sensitivity, PPV, NPV and LR + values of PNI were higher than the predictive values of albumin. Therefore, in accordance with our study’s purpose, we think that the PNI parameter is more important than the albumin parameter. PNI is used as a diagnostic indicator in malignancies, infectious diseases and cardiovascular diseases, in which inflammation is important [8,12,29]. Inflammation is very important in all complications related to diabetes [30]. This is the first study to demonstrate a relationship between PNI and amputation in DFUs. In our study, the PNI cut-off value determined for amputation was ≤39.005, and the specificity and sensitivity for this cut-off value was 93.1% and 82.7%, respectively. For SII, which is used to predict amputation in patients monitored with DFU, sensitivity is 67.4% and specificity is 63.3% [4]. PLR and NLR were evaluated for mortality in DFU, and were reported as 73.1% and 69.2% for sensitivity and as 47% and 62.6% for specificity, respectively [31]. Compared to these studies, we think that PNI is an important marker that can be used to predict amputation in DFUs, as its specificity and sensitivity are much higher in our study.

There are three limitations in the present study. The first limitation is that the study was retrospective and involved single center data. The second limitation is that the patients’ body mass indexes and diets were unknown at the time of admission to the hospital. The third limitation was that the numbers of patients with major and minor amputations were not equal. Since the number of patients with major amputation was very low, patient groups could not be divided into major and minor amputations. For PNI, it can be evaluated whether there is a significant difference between the major and minor amputee group, with a more equal and larger patient group. Despite all these limitations, our study has two important advantages. Our sample size was large when compared to previous studies with PNI. Since the patients were followed up with the same team, there was no difference in treatment approaches that may affect the amputation rate.

## 5. Conclusions

Amputation is one of the important morbidity of diabetes. Therefore, there is a need for cost-effective markers to predict amputation in patients followed up with DFU. This study showed that PNI is one of these markers. We think that patients with high risk of amputation will be noticed faster with this marker, which can be calculated in almost all health centers. In patients with malnutrition, an appropriate nutrition program can contribute to reducing the rate of amputation.

## Figures and Tables

**Table 1:** Statistical findings for the comparison of demographic and clinical characteristics of the patients between research groups.

		Non-Amputees (n = 276)	Amputees (n = 110)	*p* Values
Age, mean ± standard deviation (SD)		60.36 ± 14.26	57.77 ± 13.08	0.101 ^c^
Gender	Male	177 (64.1%)	64 (58.2%)	0.276 ^a^
	Female	99 (35.9%)	46 (41.8%)	
Smoking	No	239 (86.6%)	99 (90%)	0.360 ^a^
	Yes	37 (13.4%)	11 (10%)	
At least one comorbidity	No	107 (38.8%)	41 (37.3%)	0.785 ^a^
	Yes	169 (61.2%)	69 (62.7%)	
Rheumatological disease	No	271 (98.2%)	104 (94.5%)	0.083 ^b^
	Yes	5 (1.8%)	6 (5.5%)	
Peripheral neuropathy	No	260 (94.2%)	105 (95.5%)	0.625 ^a^
	Yes	16 (5.8%)	5 (4.5%)	
Venous insufficiency	No	274 (99.3%)	106 (96.4%)	0.058 ^b^
	Yes	2 (0.7%)	4 (3.6%)	
CAD	No	211 (76.4%)	87 (79.1%)	0.577 ^a^
	Yes	65 (23.6%)	23 (20.9%)	
CHF	No	257 (93.1%)	101 (91.8%)	0.657 ^a^
	Yes	19 (6.9%)	9 (8.2%)	
CRD	No	256 (92.8%)	101 (91.8%)	0.753 ^a^
	Yes	20 (7.2%)	9 (8.2%)	
Arterial thrombosis	No	235 (85.1%)	84 (76.4%)	0.040 ^a^
	Yes	41 (14.9%)	26 (23.6%)	
Venous thrombosis	No	268 (97.1%)	100 (90.9%)	0.009 ^a^
	Yes	8 (2.9%)	10 (9.1%)	
Osteomyelitis	No	190 (68.8%)	65 (59.1%)	0.068 ^a^
	Yes	86 (31.2%)	45 (40.9%)	

^a^ Chi square test with n (%), ^b^ Fisher exact test with n (%), ^c^ Student’s *t*-test with mean ± standard deviation (SD). CAD: Coronary artery disease, CHF: Congestive heart failure, CRD: Chronic renal disease.

**Table 2:** Statistical findings for comparison of laboratory blood values and some prognostic indicators between research groups.

	Non-Amputees (n = 276)	Amputees (n = 110)	*p* Values
WBC (10^3^/mm^3^)	8.72 (7.21–11.18)	10.78 (7.63–13.27)	0.002 ^b^
Neutrophil (10^3^/mm^3^)	5.49 (4.27–7.73)	6.66 (4.99–9.41)	0.003 ^b^
Lymphocyte (10^3^/mm^3^)	1.71 (1.23–2.12)	1.68 (1.28–2.46)	0.536 ^b^
PLT (10^3^/mm^3^)	300 (218–393)	328 (240–423)	0.135 ^b^
NLR	3.1 (2.27–4.57)	3.22 (2.42–5.86)	0.349 ^b^
PLR	17.51 (12.55–23.54)	17.81 (12.45–27.12)	0.867 ^b^
Albumin (g/L)	43 (41–44)	36 (35–38)	<0.001 ^b^
CRP (mg/L)	19.5 (10–65)	30 (8.75–114)	0.035 ^b^
Glucose (mg/dL)	133.5 (95–195)	152 (103–238.5)	0.021 ^b^
HgbA1c	7 (6–8.2)	7.4 (6.4–9)	0.015 ^b^
Urea (mg/dL)	42 (32–54)	44 (32–55.25)	0.257 ^b^
Creatinine (mg/dL)	0.9 (0.75–1.1)	0.9 (0.78–1.3)	0.300 ^b^
ESR (mm/h)	44.63 ± 28.34	50.67 ± 30.48	0.065 ^a^
SII	871 (574–1569)	1039 (607–1878)	0.184 ^b^
PNI	43.01 (41–44.02)	36.01 (35.01–38.01)	<0.001 ^b^

^a^ Student’s *t*-test with mean ± standard deviation (SD), ^b^ Mann–Whitney U test with median (Q1–Q3), WBC: White blood cell, PLT: Platelets, NLR: Neutrophil-to-lymphocyte ratio, PLR: Platelet-to-lymphocyte ratio, CRP: C-reactive protein, ESR: Erythrocyte sedimentation rate, SII: Systemic inflammatory index, PNI: Prognostic nutritional index.

**Table 3:** The findings of the ROC analysis with sensitivity, specificity, positive–negative predictive and positive likelihood ratio values demonstrating the success of the Prognostic Nutrition Index (PNI) and albumin in amputation prediction.

	Prognostic Nutritional Index (PNI)	Albumin
AUC (95% CI)	0.937 (0.911–0.963)	0.938 (0.912–0.964)
*p* values	<0.001	<0.001
Cut off	≤39.005	≤38.5
Sensitivity (95% CI)	82.7% (74.1–89)	81.8% (73.1–88.3)
Specificity (95% CI)	93.1% (89.3–95.7)	93.1% (89.3–95.7)
PPV (95% CI)	82.7% (74.1–89)	82.6% (73.9–88.9)
NPV (95% CI)	93.1% (89.3–95.7)	92.8% (88.9–95.4)
LR+ (95% CI)	12 (7.7–18.7)	11.9 (7.6–18.5)

ROC: Receiver Operating Characteristic, PPV: positive predictive value, NPV: negative predictive value, AUC: Area under curve, CI: Confidence interval, LR+: Positive likelihood ratio.

**Table 4:** The results of univariate and multivariate binary logistic regression analysis conducted to determine the risk factors that are effective in the prediction of amputation.

	Univariate		Multivariate	
	*p* Values	OR (CI 95%)	*p* Values	OR (CI 95%)
Venous thrombosis (Yes vs. no)	0.013	3.35 (1.28–8.72)	ns	-
WBC	0.006	1.00 (1.00–1.00)	ns	-
Neutrophil	0.036	1.00 (1.00–1.00)	ns	-
CRP	0.006	1.01 (1.00–1.01)	ns	-
Arterial thrombosis (Yes vs. no)	0.041	1.77 (1.02–3.08)	0.014	3.04 (1.25–7.37)
HbA1c	0.006	1.17 (1.04–1.31)	0.022	1.24 (1.03–1.48)
PNI (≤39 vs. >39)	<0.001	64.7 (32.8–127.7)	<0.001	81.8 (38.5–173.7)
Multivariate model: Nagelkerke R Square = 0.653, Classification accuracy: 90.2%				

ns: not significant (*p* > 0.05), OR: Odds ratio, CI: Confidence interval, WBC: White blood cell, CRP: C-reactive protein, PNI: Prognostic nutritional index.

## Data Availability

All data needed to support the conclusions are present in the paper. Raw data are available from the corresponding author, B.C, upon reasonable request.
